# Transcriptomic analysis of micropapillary high grade T1 urothelial bladder cancer

**DOI:** 10.1038/s41598-020-76904-7

**Published:** 2020-11-18

**Authors:** Michaela Bowden, Rosa Nadal, Chensheng W. Zhou, Lillian Werner, Justine Barletta, Nuria Juanpere, Josep Lloreta, Silvia Hernandez-Llodrà, Juan Morote, Ines de Torres, Anna Orsola, Paloma Cejas, Henry Long, Joaquim Bellmunt

**Affiliations:** 1 Department of Medical Oncology, Dana-Farber Cancer Institute, 450 Brookline Ave, Boston, MA USA; 2 Cellular and Molecular Therapeutics Branch, National Heart, Lung, and Blood Institutes, National Institutes of Health, Bethesda, MD USA; 3 Department of Biostatistics, Dana-Farber Cancer Institute, Boston, MA USA; 4 Department of Pathology, Brigham and Women’s Hospital, Boston, MA USA; 5 Department of Pathology, PSMAR-IMIM Research Institute, Barcelona, Spain; 6 Department of Health and Experimental Sciences, Universitat Pompeu Fabra, Barcelona, Spain; 7 Department of Urology, Hospital Vall D’Hebron, Universitat Autónoma de Barcelona, Barcelona, Spain; 8 Department of Pathology, Hospital Vall D’Hebron, Barcelona, Spain; 9 PSMAR-IMIM Research Institute, Barcelona, Spain; 10 Center for Functional Cancer Epigenetics, Dana Farber Cancer Institute, Boston, MA USA; 11 Department of Medical Oncology, Beth Israel Deaconess Medical Center, Harvard Medical School, 330 Brookline Av, Boston, 02215 USA

**Keywords:** Biomarkers, Medical research, Molecular medicine, Oncology

## Abstract

No consensus currently exist on the optimal treatment of patients with high-risk nonmuscle invasive (HGT1) micropapillary variant of bladder cancer (MPBC). Transcripsome analysis may allow stratification of MPBC-HGT1 enabling prediction of recurrence and guide therapeutic management for individual patients. Whole transcriptome RNA-Sequencing of tumors from 23 patients with MPBC-HGT1 and 64 conventional urothelial carcinomas (cUC) (reference set) was performed. Differentially expressed genes between MPBC-HGT1 and cUC-HGT1 were explored. Cox proportional hazard models and Kapplan–Meier methods were used to assess the relation between time to progression (TTP) and individual gene expression adjusting for clinical covariates. Over 3000 genes were differentially expressed in MPBC-HGT1 as compared with cUC-HGT1 and a 26-gene signature is characteristic of MPBC within HGT1. A set of three genes; *CD36*, *FAPB3* and *RAETE1*; were significantly associated with TTP. High expression of *FABP3* and *CD36* were associated with shorter TTP (*p* = 0.045 and *p* = 0.08) as was low expression of RAET1E (*p* = 0.01). Our study suggest that a 26-gene signature can define MPBC-HGT1 within conventional urothelial carcinomas. A prognostic risk index of three genes (*FABP3*, *CD36* and *RAET1E*) was found to be associated with shorter TTP and may help classify a group of patients with MPBC-HGT1 with high-risk of early progression. These observations might have implications in terms of radical cystectomy recommendation in MPBC patients.

## Introduction

Non-muscle invasive bladder cancer (NMIBC) is a heterogeneous disease comprising almost 75% of all bladder cancer patients^1^. Amongst several of the potential adverse features of non-invasive lesions, the presence of a variant histology (ie. micropapillary, nested, plasmacytoid, sarcomatoid) is often associated with adverse pathological features and poor outcome^2^. Micropapillary variant of bladder cancer was first described as a distinct histological subtype of bladder carcinoma in 1994^3^. This rare variant represents approximately 0.01–2.2% of urothelial tumors^1,4^ and has been associated with a higher stage at diagnosis and increased risk of metastatic disease, even if it comprises only a fraction of the overall tumor volume^5^.

Given the potential aggressive nature of HGT1 urothelial carcinoma with micropapillary features, it is currently debated whether patients with these non-invasive lesions should be offered bladder-preserving therapies using transurethral resection of the bladder tumor (TURBT) and intravesical Bacillus Calmette-Guerin (BCG) or upfront radical cystectomy (RC) that often incorporates some form of urinary diversion^6^. In the largest retrospective report of MPBC, including 44 with non-muscle invasive disease, Kamat et al.^7^ at MD Anderson found that overall prognosis was poor, and made the case for upfront RC in any HGT1 with a micropapillary component. Conversely, Spaliviero et al*.*^8^ challenged this recommendation as they observed no significant differences in 5-year disease-specific mortality or incidence of metastasis in their Memorial Sloan-Kettering Cancer Center series of 36 non-muscle invasive MPBC.

This lack of agreement might be explained by differences on the biology and molecular characteristics of MPBC^7^. There are currently only two global RNA expression studies of MPBC in MIBC^4,9,10^, which provide insigh on the intratumoral heterogeneity of bladder cancer and histologic variants, but did not established clinical recommendations based on genomic profiling.

The identification of patients at high risk for progression in MPBC-HGT1 based on molecular characteristics would help to select those who would mostly benefit from an immediate RC. In this study, we report a 26-gene signature defining of MPBC-HGT1 and a prognostic risk-index of three genes (*FABP3*, *CD36* and *RAET1E)* associated with early progression of non-invasive high-risk micropapillary variant of bladder cancer.

## Results

The clinical and tumor pathological characteristics of 87 patients with NMIBC-HGT1 are summarized in Supplementary Table 1. Twenty-three patients with MPBC-HGT1 (discovery set) and 64 cUC-HGT1 (reference set) were identified. First, we screened the transcriptome using RNA-seq and compared the gene expression profiles between patohologically annotated as MPBC-HGT1 and cUC-HGT1. More than 3000 genes were found to be differentially expressed in MP-HGT1 as compared with cUC-HGT1. The gene expression pattern for MPBC-HGT1 was more homogeneous than that of cUC-HGT1, which is known to exhibit significant inter-tumoral heterogeneity^9^.

**Figure 1:**
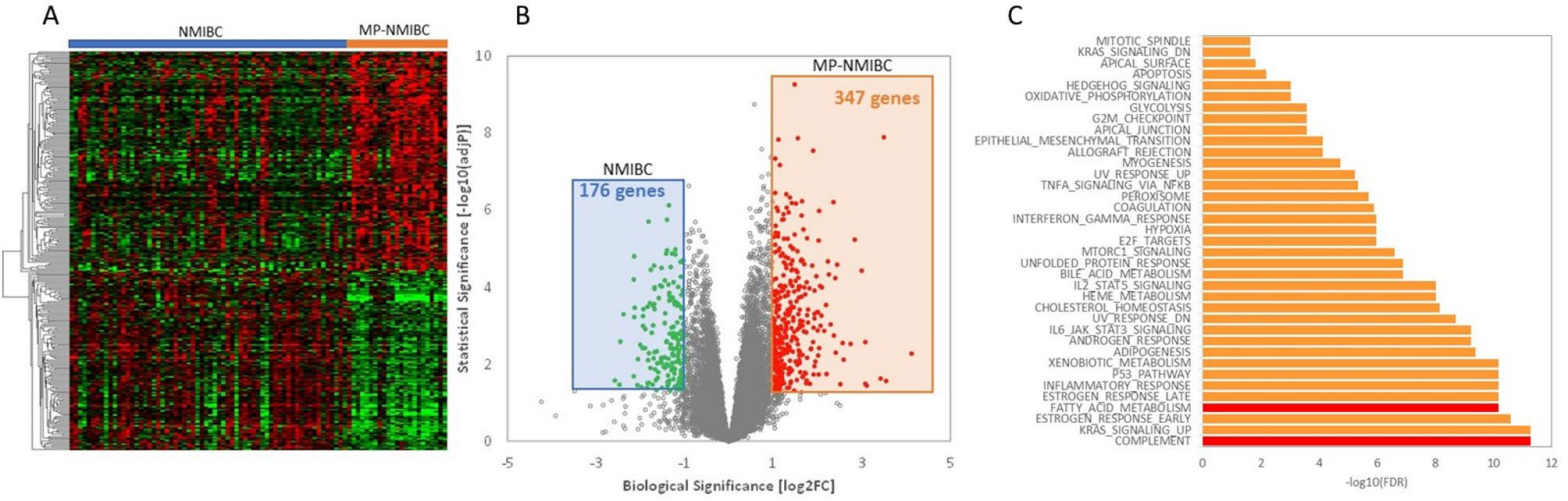
Whole transcriptome profiling identifies differentially expressed (DE) genes that define HGT1 MPBC in NMIBC tumors, where (**A**). Supervised hierarchical clustering on a heatmap separates MPBC subtype from conventional urothelial carcinoma of NMIBC. (**B**) Volcano plot illustrating fold change (logbase2) versus adjusted significant *p* value (− logbase10), where red and green data points represent a significance level of adj *p* < 0.05 and log2FC >  ± 0.6. (**C**) MSigDB Hallmark data sets enriched in DE genes, where FDR < 0.05 was significant.

**Table 1:** 26-gene signature that defines MP HGT1 MPBC in NMIBC cohort (Yes = micropapillary, No = non-micropapillary).

Gene	Description	# Patients		Median (normalized expression)		Log_2_FC	*p* value
		Yes	No	Yes	No		
COL4A4	Collagen, type IV, alpha 4	57	21	0.118	2.027	4.099	< 0.0001
LCN2	Lipocalin 2	58	23	4.057	43.073	3.408	< 0.0001
KL	Klotho	57	19	0.193	1.992	3.369	< 0.0001
ABCA13	ATP-binding cassette, sub-family A (ABC1), member 13	62	23	0.769	6.688	3.121	< 0.0001
FABP3	Fatty acid binding protein 3	61	21	2.262	14.760	2.706	< 0.0001
CYP1B1	Cytochrome P450 family 1 subfamily B member 1	64	23	0.028	0.125	2.168	< 0.0001
ANPEP	Alanyl aminopeptidase	63	23	1.067	2.960	1.472	< 0.0001
DACH2	Dachshund family transcription factor 2	64	23	0.053	0.004	− 3.585	< 0.0001
KRTAP5-9	Keratin-associated protein 5-9	64	23	0.355	0.022	− 4.018	< 0.0001
CAPNS2	Calpain small subunit 2	64	23	0.618	0.021	− 4.887	< 0.0001
KRTAP5-8	Keratin-associated protein 5-8	64	23	0.420	0.009	− 5.576	< 0.0001
NPAP1	Nuclear pore associated protein 1	64	23	0.040	0.001	− 5.637	< 0.0001
GDPD2	Glycerophosphodiester phosphodiesterase domain containing 2	64	23	0.212	0.004	− 5.689	< 0.0001
HECTD2-AS1	HECTD2 antisense RNA 1	64	23	0.091	0.000	− 9.827	< 0.0001
SNHG8	Small nucleolar RNA host gene 8	64	23	0.114	0.000	− 10.148	< 0.0001
GABBR2	Gamma-aminobutyric acid (GABA) B receptor, 2	57	23	0.484	6.182	3.675	0.0001
C12orf75	Chromosome 12 open reading frame 75	61	21	3.689	20.363	2.465	0.0003
PKP1	Plakophilin 1	64	23	0.364	0.055	− 2.727	0.0006
CEACAM6	Carcinoembryonic antigen-related cell adhesion molecule 6	57	23	2.124	9.993	2.234	0.0009
CRTAC1	Cartilage acidic protein 1	63	18	12.147	0.983	− 3.627	0.0014
CD36	Platelet glycoprotein IV	64	23	0.128	0.683	2.421	0.0032
IGF2	Insulin-like growth factor 2	64	23	3.876	0.483	− 3.004	0.0038
MMP7	Matrix metallopeptidase 7	51	21	1.847	15.275	3.048	0.0085
RAET1E	Retinoic acid early transcript 1E	60	10	4.012	1.036	− 1.954	0.0124
CHST3	Carbohydrate sulfotransferase 3	64	23	0.820	1.530	0.900	0.0394
TNFRSF11B	TNF receptor superfamily member 11b	64	23	0.032	0.084	1.378	0.0662

Upon application of cutoffs (± twofold change, Adj *p *value < 0.05), this analysis revealed 523 genes to be differentially expressed between MPBC-HGT1 and cUC-HGT1 (Fig. 1A). A volcano plot showed the most up- and down-regulated genes, factoring in both biological and statistical significance (Fig. 1B).

We performed pathway enrichment analysis using the MSigDB Hallmark gene set and plotted the statistically significant (FDR < 0.05) enriched gene sets that were found to be upregulated in MPBC-HGT1 (Fig. 1C). We found that MPBC-HGT1 were enriched with expression signatures involved in immune system, cell cycle, metabolic pathways and targets, of which the most enriched hallmark gene set was the complement pathway. To define a minimal set of genes that could accurately classify MP-HGT1, we used biological and statistical cutoffs of twofold differential expression and adjP value less than 0.05. We derived a 26-gene signature defining of MPBC-HGT1 (Table 1). This 26-gene signature was enriched for genes associated with metabolism and metabolic transport, immune response, extracellular matrix and osteoblast differentiation.

**Figure 2:**
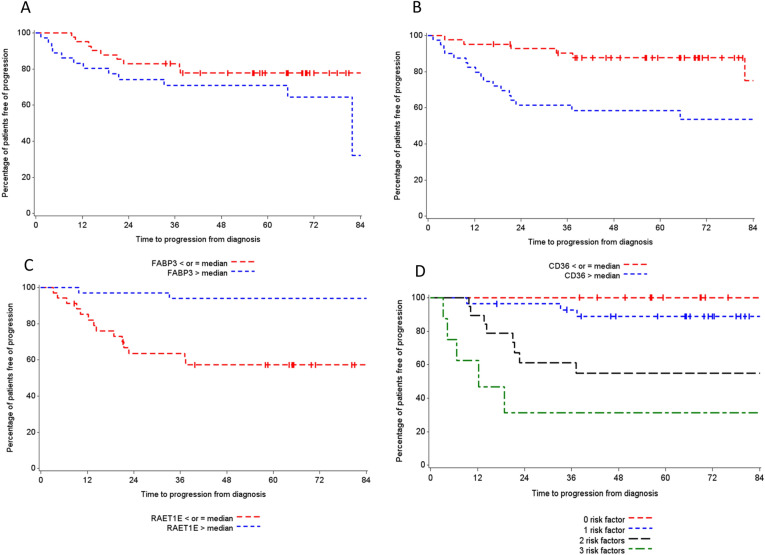
Kaplan–Meier survival product limit estimates for NMIBC patients with micropapillary variant for CD36 (**A**), RAET1E (**B**) and FAPB3 (**C**) transcript levels and in combination (see text for risk factor description) (**D**). The *p *value was calculated using the log-rank test between patients with high (> median) and low (≤ median) expression.

In an attempt to understand which individual genes of the MP-HGT1 defining 26-gene signature are more representative in evaluation of clinical outcome, Kaplan–Meier curves (log-rank test) and Cox regression analysis were use to evaluate the association between individual GE and TTP. Individual GE levels were dichotomized into low and high using the overall patient median GE as the cutoff (Table 2). Individual genes that showed a statistically significant association with TTP or generated Kaplan–Meier curves highlighting a clear separation with respect to progression are shown in Fig. 2. We found that high *CD36* expression (*p* = 0.002, Fig. 2B) and low expression of *RAETE1* (*p* = 0.0005, Fig. 2C) were associated with shorter TTP in univariate analysis. There was trend of higher expression of FABP3 (*p* = 0.13, Fig. 2A) associated with shorter TTP. Multivariate analysis confirmed that high expression of *FABP3* was independently associated with shorter TTP (HR: 0.33; 95% CI 0.11–0.97; *p* = 0.045); as was low expression of *RAET1E* (HR: 14.14; 95% CI 1.74–114.85; *p* = 0.01) in the entire HGT1 cohort. High expression of *CD36* was correlated with a trend towards shorter TTP, though this did not reach statistical significance (HR: 0.31; 95% CI 0.08–1.15; *p* = 0.08).

**Table 2:** Univariate and multivariate analyses of FABP3, CD36 and RAET1E association with TTP in NMIBC micropapillary variant cohort.

	Expression	Progression	Univariate analysis			Multivariate analysis		
			HR	95% CI	*p* value	HR	95% CI	*p* value
FABP3	High	12/36	1 (ref)			1 (ref)		
	Low	9/41	0.56	0.21–1.49	0.13	0.33	0.11–0.97	0.045
CD36	High	18/40	1 (ref)			1 (ref)		
	Low	6/42	0.20	0.06–0.61	0.002	0.31	0.08–1.15	0.08
RAET1E	High	2/34	1 (ref)			1 (ref)		
	Low	14/34	0.86	1.88–36.40	0.0005	14.14	1.74–114.85	0.01

Expression was defined as high (> Median) or low (≤ Median).

Additionally, we generated a new high/low risk index to combine *FABP3, CD36* and *RAETE1.* Patients were categorized into a high-risk group for progression when tumor had high expression levels of *FABP3* and *CD36* and low expression of *RAETE1.* The new index assigns the expression of any of these 3 genes, as a risk factor of 1, any 2 of the 3 genes, a risk factor of 2, and the combined expression of all 3, a risk factor of 3. Increasing risk factor was associated with shorter TTP (Table 3). A combination of all 3 genes was significantly associated with a shorter TTP (*p* < 0.0001) in the univariate analysis (Fig. 2D).

**Table 3:** Univariate analysis for 3-gene combination of CD36 (high expression), FAPB3 (high expression) and RAETE1 (low expression) transcript levels association with time to progression in NMIBC cancers patients with micropapillary variant.

	Progression	Univariate analysis		
		HR	95% CI	*p* value
3-gene combination (FABP3, CD36, RAETE1)				< 0.0001
0	0/11	0	0	
1	3/28	0.07	0.02–0.31	
2	8/19	0.38	0.13–1.18	
3	5/8	1 (reference)		

### Correlation with the 5 RNA signature subtypes described in The Cancer Genoma Atlas (TCGA) 2017

**Figure 3:**
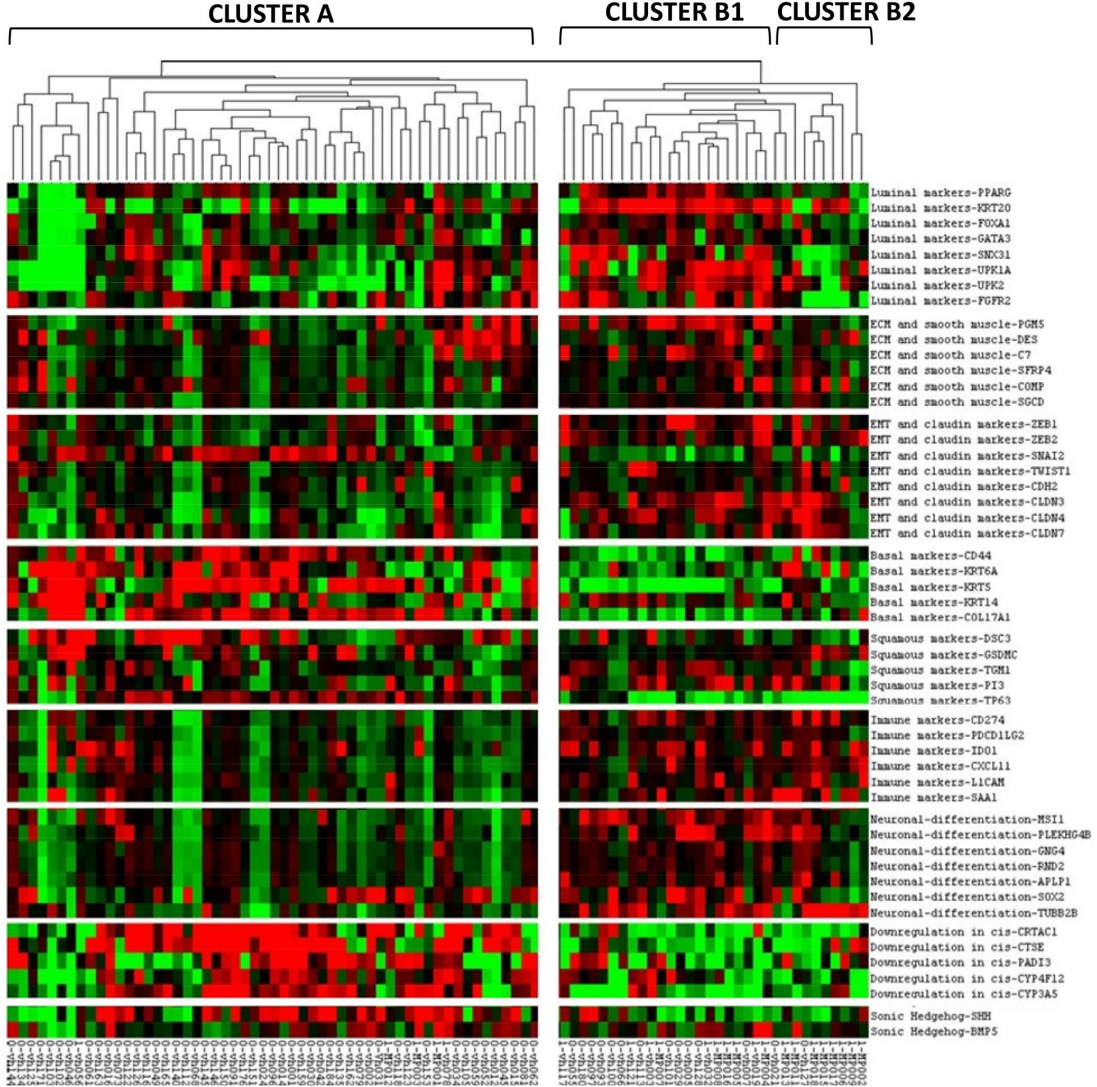
MP variant in NMIBC distribution across the 5 major bladder cancer molecular subtypes; luminal-papillary, luminal-infiltrated, luminal, basal-squamous and neuronal.

Recently discriminated subtypes from more than 413 MIBC tumor specimens, by Robertson et al.^11^, comprising the luminal-papillary, luminal-infiltrated, luminal, basal-squamous and neuronal molecular subtypes, expanded upon previous studies^12–14^ in the TGCA 2014. We took the gene signature that defines the 5 TCGA subtypes and conducted unsupervised clustering of the RNA-Seq data from our HGT1 cohort (n = 87) to establish where the MPBC-HGT1 tumors would align (Fig. 3) within the 5 described TCGA 2017 subtypes^11^.

In our HGT1 cohort, we observed 2 major Clusters, A (64%, 56/88) and B (36%, 32/88) with some differentiation within B, B1 (25%, 22/88) and B2 (11%, 10/88). The majority of tumors in Cluster A showed low or no expression of immune and neuronal genes. There was also a lack of Epithelial Mesenquimal Transition (EMT) genes expressed in this cluster, which represent the luminal-infiltrated molecular subtype, so Cluster A could be considered a less aggressive phenotype based on Robertson gene classification. This could be equivalent to GS1 in the Uromol paper^15^. Approximately 70% of tumors in Cluster A had moderate/high expression of basal-squamous markers, with the remaining 30% of tumors showing moderate/high expression of luminal and luminal-papillary markers. Cluster B was enriched for tumors where high expression of the 3 luminal molecular subtypes, luminal (53%), luminal-papillary (66%) and luminal-infiltrated (66%), coupled with the low/absence of expression of the basal-squamous subtype was observed. In contrast to Cluster A, immune and neuronal genes had moderate/high expression. Cluster B2 was differentiated from Cluster B1 based on lower expression of the luminal molecular subytpe genes, such as *PPARG*, *FOXA1*, *SNX31* and *FGFR2* in the latter. Cluster B could be considered a more aggressive phenotype than Cluster A based on Robertson gene classification^11^ with the most prominent aggressive markers (infiltrated-luminal) more highly expressed in Cluster B2. This B2 cluster correlates with the Ga in Uromol^15^ As expected, Cluster B1 was enriched for HGT1 NMIBC tumors harboring the MPBC variant (44%, 10/23), followed by Cluster B2 (34%, 8/23) and Cluster A (22%, 5/23).

Recently Guo et al.^10^ published a gene expression profiling study characterizing the micropapillary variant in MIBC. In that study, a 20-gene signature defining the MPBC-MIBC phenotype was shown to be associated with worse overall survival. Hierarchical clustering of the NMIBC and MPBC-NMIBC using the Guo signature was undertaken (Supplementary Fig. 1S). A MPBC-HGT1 enriched cluster was evident, however only 3 of the 20 genes reached threshold of biological [± twofold change] and statistical significance [Adj *p* < 0.05]. *CLDN3* (FC = 2.0592; *p* = 3.3 × 10^–5^), *GPPD3* (FC = 2.0154; *p* = 0.0025) and *MUC1* (FC = 2.7191, *p* = 0.0209) were all upregulated in MP-HGT1 compared to cUC-HGT1, which recapitulated the Guo et al. findings. In addition, we conducted univariate analysis for the 20 genes and found that only 2 genes, *KCNF1* and *LY6D* were associated with TTP in our cohort of MPBC-HGT1. Low expression of *KCNF1* (*p* = 0.03) and *LY6D* (*p* = 0.008) were associated with shorter TTP by univariate analysis.

Hedegaard et al.^15^ identified in 460 NMIBC (that included 59.1% with low-grade tumors) 3 major transcriptomic clusters, representing luminal (Class 1), luminal-infiltrated (Class 2) and basal (Class 3) molecular subtypes, which generally reflect clusters as have been previously defined for MIBC. Class 1 and 2 are comprised of genes with luminal-like characteristics and are distinguished from each other based on diverging aggressivity, where Class 1 represents the luminal subtype and Class 2 the luminal-infiltrated subtype, associated with a worse prognosis. Class 3 represents the basal subtype, associated with lower grade and lower stage of disease. Based upon a validated 117-gene classifier that defined these subtypes, we conducted unsupervised clustering of the RNA-Seq data from our HGT1 cohort (n = 87) to establish where the MPBC-HGT1 tumors would align (Supplementary Fig. 2S). Fifty percent of the HGT1 cohort clustered in the most aggressive Class 2 luminal-infiltrated subtype, followed by 31% in Class 1 luminal subtype and 19% in the best prognostic Class 3 basal subtype. The Class 2 subtype was also enriched for MP-HGT1 (75%, 17/23), compared with Class 1 (22%, 5/23) and Class 3 (only 4%, 1/23).

### *FABPS* and CD36 quantitative mRNA expression analysis in the independent cohort

To validate the microarray expression data, we performed quantitative real-time RT-PCR (qPCR) to assess *FABP3* and *CD36* gene expression in an additional set of 11 MP-HG (6 HGT1 and 5 HG T2/4). Expression levels were classified in 3 categories: low, moderate or high.There was a statistically significant difference (*p* = 0.01) in *FABPS* expression between MP-HGT1 (median of 0.016 (0.007–0.025) and MP-HG T2/4 [median of 0.002 (0.001–0.012)], but not in *CD36* expression (Supplementary Fig. 3S). With a median follow-up of 4 years, 3 out of 6 HGT1 patient had early recurrence (cases #1 to #3) and all had either moderate or high expression levels of *FABP3* or *CD36* (Supplementary Table 2). Technical limitations of RT-PCR experiments prevented the validation of *RAET1E* by qPCR.

## Discussion

There is no question that RNA-based subtyping has helped in deconvoluting some of the heterogeneity of bladder cancer^7,13–15^. Our study shows that the micropapillary variant in HGT1 has a distinct transcriptomic profile from that of cUC HGT1. More than 3000 genes were found to be differentially expressed, representing a diverse set of enriched pathways encompassing immune, metabolic and cell cycle/apoptosis genes. From this, we identified a 26-gene signature that defined the MP variant and found that high expression of *CD36* and *FABP3* and low expression of *RAET1E* were associated with shorter TTP.

The MPBC is a variant histology that has been linked to aggressive behavior and a lack of BCG response has been speculated^7,16^. Prospective trials are missing due to the rarity of this variant, and recommendations on whether to proceed to immediate RC or to follow the standard approaches used in HGT1 tumors are contradictory. In addition to the well-characterized clinicopathologic criteria to predict recurrence and progression^17^, RNA expression analysis might help identify patients with MPBC variant that portent highly aggressive behavior and a poor outcome in HGT1 patients. Identifying patients with poor outcome will help to recommend RC as first-line therapy and reserve induction BCG for patient with favorable prognosis genomically identified tumors.

FABP3 plays various roles in fatty acid transport, cell signaling, cell growth, and gene transcription^18^ as well as, tumorigenesis^19^. Furthermore, in vitro inhibition of FABP3 inhibits cell proliferation and impairs tumor growth^20^. *FABP3 overexpreesion* has also been implicated in driving unfavorable prognosis in other maligancies suh as gastric cancer^21^ and non-small cell lung carcinoma^19,22^. *CD36*, also known as fatty acid translocase, is associated with fatty acid uptake interacting with lipoproteins and long-chain fatty acids, such as *FABP3.* Previous studies have shown that overexpression of *CD36* results in elevated fatty acid uptake and promotion of a more aggressive epithelial-mesenchymal transition (EMT) phenotype in a number of cancers, including hepatocellular carcinoma^23^. Gene expression markers, such as *ZEB1*, *ZEB2*, *SNAIL2*, *TWIST 1*, *CDH2*, *CLDN3*, *CDLN4*, *CLDN7*, describe EMT-like characteristics that distinguishes the infiltrated-luminal subtype from the luminal and papillary-luminal subtypes. In large-scale reported studies, high expression of EMT-related genes confers worse outcome in both MIBC^11^ and NMIBC^15^. Our findings may underline a biologic basis for why at least a subset of MB-HGT1 have a more aggressive clinical behavior and require a more aggressive treatment approach.

Retinoic acid early transcripts, encoded by RAET1 genes are a family of ligands for NKG2D in humans which are frequently expressed by tumor cells and participate in natural killer (NK) cells mediated anticancer immune response. Diverse lines of evidence suggest that the expression of NKG2D ligands predicts prognostic in different malignancies^24^. Our work suggests that low expression of *RAET1* is associated with poor outcomes suggesting and involvement of NKG2D-mediated immunity in micropapillary HGT1 bladder cancers.

We could not reproduce the prognostic 20-gene signature defining the MPBC-MIBC phenotype reported by Guo et al*.* in our dataset. We hypothesize that comparing muscle-invasive bladder cancer cases in Guo et al*.* cohort with ours which only include HGT1 cases may account for the differences observed. Hedegaard et al.^15^ series is biased to low-risk NMIBC as less than 25% of cases were HGT1. Our HGT1 cohort clustered with the most aggressive Class 2 luminal-infiltrated subtype enriched in patients harboring the MP variant (75%, 17/23). By utilizing the Robertson^11^ MIBC signature, we found that the HGT1 cohort was enriched in the 3 luminal subtypes with a very small representation in the basal-squamous subtype consistent with recently reported data^9^. We also evaluated a reported MPBC variant gene signature derived in a MIBC cohort^10^. Although some trends, where *CLDN3*, *GPPD3* and *MUC1* were significant in terms of the differentially expressed genes in the MPBC-HGT1, ultimately we only found 2 genes, *KCNF1* and *LY6D* that were associated with TTP. Of note, we observed that the association with *LY6D* was expressed (low) in the opposite direction to the signature as derived for MIBC.

With these additional analyses, we did not identify the 5 TCGA molecular classes for MIBC in the HGT1 cohort and we were not able to replicate the prognostic 20-gene signature identified in MPBC-MIBC either^10,11^. Coupled with the unique 3-gene signature we found in our MPBC HGT1 cohort, it would appear that the MPBC variant within NMIBC may harbor properties of the MPBC variant in more advance disease stages or grades but requires a unique molecular classification to determine which patients are likely to progress to a more aggressive phenotype. MPBC variant in NMIBC is also predominantly of luminal subtype, but interestingly, we found that MPBC variant in HGT1 was also enriched for immune and neuronal subtypes. Our group opted for the TCGA 20017 classifier as the methodology used was in line with our experimental approach rather than the 2019 Consensus Classification that included a mixture of RNA sequencing and microarray technology in some of the data sets analyzed^25^.

Our findings confirm that MPBC variant has a unique and characteristic gene expression signature that makes this variant histology entirely different among the more conventional urothelial carcinoma histology. This different molecular profile supports the rational for a different biological pathway for the MPBC variant. Limitations of the present analysis include the lack of specific quantification of the percentage of MPBC tumor > 10% in mixed tumors and the limited sample size.

In our HGT1 cohort, the MPBC variant histology alone was not an adverse prognostic factor per se (data not shown), and we did observe that only a subgroup with MPBC characterized by differentially expressed genes had poor outcome. We found that 3 DE genes could help to discriminate MP for poor outcome. High expression of *FABP3* and low expression of *RAET1E* were independently associated with shorter TTP with a trend of high expression of *CD36*. A risk index generated by combining *FABP3*, *CD36* and *RAETE1* was able to link increasing risk factor score with shorter TTP. Although further validation before this three-gene prognostic classifier is suitable for clinical implementation, this data set is key to our understanding of the disease, and findings derived from it can be applied to additional independent cohorts.

Our findings highlight variable genomic heterogeneity existing across the different stages, and variant histologies of bladder cancer. Preliminary confirmation of the value of *FABP3* and *CD36* was observed in an small independent data set of patients with MPBC-HGT1. While awaiting further independent validation in a larger data set, this study provides important insights on the underlying differential biology of MPBC variant histology. Analysis of 3 differentially expressed genes has allowed to create a risk index that might help to indentify patient with MPBC-HGT1 with a higher risk of progression in which RC could be recommended as a first treatment option.

## Conclusions

Our study provides a comprehensive insight into the transcriptome of non-muscle invasive micropapillary variant of bladder cancer. We identified a set of 523 genes which are differentially expressed genes between MP- and cUC-NMIBC and a 26-gene signature that characterize MPBC-NMIBC within conventional urothelial carcinomas. A prognostic risk-index of three genes (*FABP3*, *CD36* and *RAET1E)* was developed to predict time to progression. This classified a high-risk group of patients with MP-NMIBC who are at risk of early disease progression and most likely would benefit from agressive surgical management. This molecular-based approach would limit the generalizability of up-front cystectomy for all MPBC-NMIBC patients.

## Methods

### Clinical cohort

Two hundred primary urothelial HGT1 cases consecutively diagnosed in two different centers (Hospital del Mar (HM), MARBiobanc, Barcelona, Spain and Hospital Vall d’Hebron (HVH), Barcelona, Spain) were included in this study between April 2004 and April 2011^26^. Archival FFPE tissue specimens were collected at the time of TURBT confirming the diagnosis of MPBC-NMIBC. Only patients whose tumors yielded a high-quality total RNA suitable for array analysis were included in this study (n = 87). Twenty-three patients with MPBC-HGT1 (discovery set) and 64 cUC-HGT1 (reference set) were identified.

The median follow-up of the entire cohort was 7.4 years. Patients were managed uniformely using TURBT and intravesical BCG therapy. Patients with T1b substaging underwent re-TURBT after BCG therapy as per institutional approved protocol^26^. (Supplementary Methods) Clinical outcome was classified as: non-recurrent disease, recurrent disease or progression to muscle invasive disease or metastasis ≤ 4 years. Time to progression (TTP) was defined as the time interval from initial pathological diagnosis of HGT1 until the time to progression to MIBC, metastasis or death from BC. Informed consent was provided by each subject and use of the tissue was approved by the Ethics Committee of the Dana Farber Cancer Institute, HM and the HVH and all research complied with local ethics guidelines.

### Pathology review and annotation

In all cases, 4 μM hematoxylin–eosin-stained histologic preparations were available for review. Parameters reviewed included tumor location, percentage of MPBC, tumor cell grade, depth of tumor invasion, lymphovascular invasion, presence of concurrent conventional papillary noninvasive or invasive urothelial carcinoma and urothelial carcinoma in situ, and presence of glandular, squamous, or another differentiation of urothelial carcinoma.

Tumors were graded according to the 2004 WHO system (2006) after pathological assessment of the total specimen. A two tier system^27^ was used to assess the depth of lamina propria (LP) invasion: T1a when tumor involved the subepithelial connective tissue superficial to muscularis mucosae (MM); T1b when tumor was found at the level of or beyond MM. For the MPBC-HGT1, only tumors with at least a > 10% of MP component measured by a semiquantitative (visual) estimation of the micropapillary component percentage were included^28^. Only initial HGT1 tumors with a visible, clearly identifiable and disease-free muscularis propria and MPBC-HGT1 on which both pathologists concur on the presence of MP component (> 10%) were included in this study. No other histological variants were included in this analysis.

Pathology review of tumor regions of interest (ROI), where tumor cellularity was in excess of 70% were annotated by two pathologists (JBa, NJ). Up to 5 × 0.6 mm cores were punched from the FFPE tissue blocks within the tumor-rich ROI. Every effort was made when sampling micropapillary areas to ensure that micropapillary histology was present throughout the thickness of the tissue core.

### RNA extraction, QC and quantification from archival FFPE tissue specimens

The AllPrep FFPE Kit (Qiagen, Germantown, Maryland, USA) was utilized for the RNA extraction from archival FFPE samples for RNA sequencing in this study. RNA isolates were eluted in a 14 μl volume of RNase/DNase-free H_2_O. RNA was quantified utilizing the Quant-iT RiboGreen Assay (Life Technologies—cat# R11490).

RNA quality was assessed on Agilent Bioanalyer using RNA 6000 Pico or nano kit. Since RNA from FFPE are all degraded, DV200 analysis were performed instead of RIN (RNA integrity number) for a more accurate read out of RNA quality. DV200 is an analysis recommended by the RNA seq kit protocol, which calculates the percentage of RNA fragments larger than 200 nucleotides. A higher DV200 score indicates better RNA quality. For library preparation, varies quantity of RNA were used to compensate for quality variations following the Library construction kit (Illumina RNA Exome kit) instructions. For RNA with DV200 higher than 70%, 20 ng RNA was used for library construction; for DV200 between 50–70%, 40 ng; and for DV200 under 50%, 100 ng^29^. All RNA library passed quality assessment before putting into whole transcriptome hybridization and capture. Post-capture libraries passed QC on TapeStation before loading into sequencer. Samples that disqualify were dismissed.

### Automated RNA-Seq library preparation and sequencing

50 ng input total RNA was utilized for RNA-Seq library preparation utilizing the TruSeq Stranded RNA Access Library Prep Kit (cat# RS-301-2001). The method was automated on the Biomek FXP Laboratory Automation Workstation (Beckman Coulter). cDNA libraries were quantified utilizing the Quanti-iT PicoGreen assay (Life Technologies—cat# P7589). Libraries were also quality checked by Agilent Bioanalyzer using the High Sensitivity DNA kit (cat# 5067-4626). cDNA libraries were sequenced on the Illumina NextSeq500 platform as 75 bp paired end reads. The STAR RNA sequencing alignment tool (Spliced Transcripts Alignment to a Reference) aligner [STAR_2.5.0a]) was utilized to align the data to the genome, Homo sapiens UCSC hg19 (RefSeq gene annotations). DeSeq2 was used to perform differential expression analysis (± twofold, adj *p* value < 0.05). We computed overlaps between our differentially expressed gene set and the annotated Hallmark gene sets in the Molecular Signature DataBase, MSigDB (top 100, FDR *q* value < 0.05). Gene Cluster 3.0 was used to perform hierarchical clustering. Java TreeView (Version 1.1.5) software was used to plot the heatmap from hierarchical clustering results. The material presented here is original research, mRNA data has been uploaded at GEO (GEO: GSE136401. Token for reviewers: gzazgmomxdglnwb).

### *FABPS* and *CD36* quantitative mRNA expression analysis (qPCR)

Total RNA was extracted in an additional set of 11 MPBC-NIMBC from FFPE (MARBiobanc, Barcelona, Spain) with the RNeasy Mini kit (Qiagen, Cathsworth, CA, U.S.A.). According to stage and grade classification tumors were: 6 HGT1 and 5 HGT2-T4 tumors. cDNA was synthesized using 1 μg of total RNA and SuperScript IV VILO with ezDNase (Thermo Fisher Scientific, Carlsbad, CA, USA) according to the manufacturer’s instructions.

*FABPS* and *CD36* mRNA expression were analyzed by quantitative Real-Time PCR (qPCR) in all MPBC samples with the ABI PRISM 7500 Sequence Detection System, using the TaqMan Gene Expression Assay probe and primer mix (Applied Biosystems, Life Technologies Corporation, CA, USA). The assay identification number for *FABPS* and *CD36 *were Hs00997360_m1 and Hs00354519_m1, respectively. *GAPDH* (4310884E) gene was used as internal control to normalize levels of mRNA expression. Samples were run in triplicate and the mean value was calculated for each case. The 2^(− ΔCt)^ of *FABP3* and *CD36* versus *GAPDH* was applied to normalize levels of expression in each sample. The relative expression of *FABP3* in MPBC samples ranged between 0.0012 and 0.026. According to *FABP3* expression levels, 3 groups were stablished: low expression with values from 0.0012 to 0.0032; moderate expression with values from 0.0075 to 0.013; and high expression for values > 0.025. The relative expression of *CD36* in MPBC samples ranged between 0.0003 and 0.012. According to *CD36* also expression levels, 3 groups were stablished: low expression with values from 0.0003 to 0.0006; moderate expression with values from 0.0007 to 0.0009; and high expression for values > 0.002.

### Statistical analyses

Patient and clinical characteristics were summarized as numbers and percentages. The Wilcoxon rank sum test was used to test for significant associations between dichotomous clinical and pathological variables, TTP and gene expression. TTP probabilities were estimated using the Kaplan–Meier method, and the log-rank test was used to determine the level of significance between survival curves. Cox regression analysis was used to assess the association between gene expression and survival while controlling for microstaging (pT1a vs pT1b)^30^ and micropapillary pattern. Hazard ratios and 95% confidence interval (CI) were reported as well.

## Supplementary information

Supplementary Information.
