# Supplementary material for: Transcriptomic analysis of micropapillary high grade T1 urothelial bladder cancer
Source: Sci Rep. 2020 Nov 18;10:20135. doi: 10.1038/s41598-020-76904-7 (PMC7675970; doi:10.1038/s41598-020-76904-7)
Supplement: Supplementary file 1 — Supplementary Information. [file 41598_2020_76904_MOESM1_ESM.docx]

**Supplementary Data**

**Transcriptomic Analysis of Micropapillary High Grade T1 Urothelial Bladder Cancer**

Michaela Bowden^1*^, Rosa Nadal^2*^, Chensheng W Zhou^1^, Lillian Werner^3^, Justine Barletta^4^, Nuria Juanpere^5,6^, Josep Lloreta^5,6^, Silvia Hernandez-Llodrà^6^,Juan Morote^7^, Ines de Torres^8^ , Anna Orsola^9^, Paloma Cejas^9^, Henry Long^10^, Joaquim Bellmunt^1,9,11^

^1^Department of Medical Oncology, Dana-Farber Cancer Institute Boston, MA, USA.

^2^Cellular and Molecular Therapeutics Branch. National Heart, Lung, and Blood Institutes, National Institutes of Health, Bethesda, MD, USA.

^3^Department of Biostatistics, Dana-Farber Cancer Institute, Boston, MA, USA.

^4^Department of Pathology, Brigham and Women’s Hospital, Boston, MA, USA.

^5^Department of Pathology, PSMAR-IMIM Research Institute , Barcelona, Spain.

^6^Department of Health and Experimental Sciences, Universitat Pompeu Fabra, Barcelona, Spain

^7^ Department of Urology, Hospital Vall d’Hebron, Universitat Autónoma de Barcelona, Spain.

^8^Department of Pathology, Hospital Vall d’Hebron, Barcelona, Spain.

^9^PSMAR-IMIM Research Institute, Barcelona, Spain.

^10^Center for Functional Cancer Epigenetics. Dana Farber Cancer Institute, Boston, MA, USA.

^11^Department of Medical Oncology, Beth Israel Deaconess Medical Center. Harvard Medical School. Boston, USA.

**Supplementary Methods**

**Supplementary Figures S1-S3**

**Supplementary Tables S1-S2**

**Supplementary Methods**

Patients diagnosed at initial transurethral resection (TUR) with HGT1 bladder cancer were enrolled in a clinical trial named: ’Optimized Treatment Strategy for HighGrade1 (HGT1) Bladder Cancer Based on Substaging: A Prospective Observational Cohort Study. (NCT02113501).

Transurethral resection of the bladder tumor (TURBT) included complete resection of all visible tumors, with a separate base biopsy. Cold cup biopsies to detect CIS were taken in a standardized manner or according to EAU Guidelines depending on surgeon preference (abnormal urothelium, non-papillary tumor appearance, or positive cytology) ^1^. All patients received a postoperative dose of intravesical mitomycin-C.

Patients willing to participate

received 6 weekly instillations of 2–8

×

10

8

colony-forming units Tice BCG (OncoTICE®)

and were then assigned according to MM

involvement to undergo, at 3 months after

the initial TUR and BCG induction, either a

postBCG-TUR (in the case of T1b disease) or a

cystoscopy plus cytology (T1a cases). T1a

cases were assigned to cystoscopy and

cytology based on the lower risk of both

progression and understaging [15,16] and

underwent a postBCG-TUR or bladder

biopsies only if ﬁndings were positive. The

postBCG-TUR included resection of the

bladder scar, resection of any visible tumour

and random bladder biopsies if CIS had been

identiﬁed initially.

Patients willing to participate

received 6 weekly instillations of 2–8

×

10

8

colony-forming units Tice BCG (OncoTICE®)

and were then assigned according to MM

involvement to undergo, at 3 months after

the initial TUR and BCG induction, either a

postBCG-TUR (in the case of T1b disease) or a

cystoscopy plus cytology (T1a cases). T1a

cases were assigned to cystoscopy and

cytology based on the lower risk of both

progression and understaging [15,16] and

underwent a postBCG-TUR or bladder

biopsies only if ﬁndings were positive. The

postBCG-TUR included resection of the

bladder scar, resection of any visible tumour

and random bladder biopsies if CIS had been

identiﬁed initially.

Enrolled subjects received six weekly instillations of 2–8x10e8 UFC BCG Tice (OncoTICE, Schering-Plough Canada Inc., Kirkland, QC, Canada). At 3 months after the TURBT and BCG induction, either postBCG-TUR (in the case of T1b disease) or a cystoscopy plus cytology (T1a cases). T1a cases were assigned to cystoscopy and cytology based on the lower risk of both progression and understaging and underwent a postBCG‐TUR or bladder biopsies only if findings were positive. The postBCG-TUR included reseaction of the bladder scar, resection of any visible tumor and random bladder biopsies if CIS had been identified initially.^2^

and were then assigned according to MM

involvement to undergo, at 3 months after

the initial TUR and BCG induction, either a

postBCG-TUR (in the case of T1b disease) or a

cystoscopy plus cytology (T1a cases

Cases with a negative evaluation after the BCG induction continued on BCG maintenance for 3 years and were followed with cystoscopy and cytology every 6 months. For all patients with positive findings at 3 months, the final decision about treatment, including the option of cystectomy, was taken according to disease status and the patient’s preference.

**REFERENCES**

1 Babjuk, M. *et al.* EAU guidelines on non-muscle-invasive urothelial carcinoma of the bladder: update 2013. *Eur Urol* **64**, 639-653, doi:10.1016/j.eururo.2013.06.003 (2013).

2 Orsola, A. *et al.* Reexamining treatment of high-grade T1 bladder cancer according to depth of lamina propria invasion: a prospective trial of 200 patients. *British journal of cancer* **112**, 468-474, doi:10.1038/bjc.2014.633 (2015).

**Supplementary Table S2**

*FABPS* and *CD36* quantitative gene expression in MPBC by qPCR.

| **Case** | **TNM** | ***FABP3* qPCR 2^(-∆Ct)** | | ***CD36* qPCR 2^(-∆Ct)** | |
| --- | --- | --- | --- | --- | --- |
| **#1** | HGT1 | 0,0075 | MODERATE | 0,0023 | HIGH |
| **#2** | HGT1 | 0,013 | MODERATE | 0,0009 | MODERATE |
| **#3** | HGT1 | 0,014 | HIGH | 0,0042 | HIGH |
| #4 | HGT1 | 0,019 | HIGH | 0,0006 | LOW |
| **#5** | HGT1 | 0,025 | HIGH | 0,0003 | LOW |
| **#6** | HGT1 | 0,026 | HIGH | 0,0053 | HIGH |
| **#7** | HGT2 | 0,0012 | LOW | 0,0005 | LOW |
| **#8** | HGT3 | 0,0012 | LOW | 0,0004 | LOW |
| **#9** | HGT3 | 0,013 | MODERATE | 0,0037 | HIGH |
| **#10** | HGT4 | 0,0023 | LOW | 0,0007 | MODERATE |
| **#11** | HGT4 | 0,0032 | LOW | 0,012 | HIGH |
